# Blood Mercury Levels in Children with Kawasaki Disease and Disease Outcome

**DOI:** 10.3390/ijerph17103726

**Published:** 2020-05-25

**Authors:** Ling-Sai Chang, Jia-Huei Yan, Jin-Yu Li, Deniz Des Yeter, Ying-Hsien Huang, Mindy Ming-Huey Guo, Mao-Hung Lo, Ho-Chang Kuo

**Affiliations:** 1 Department of Pediatrics and Kawasaki Disease Center, Kaohsiung Chang Gung Memorial Hospital and Chang Gung University College of Medicine, Kaohsiung 83301, Taiwan; joycejohnsyoko@gmail.com (L.-S.C.); yhhuang123@yahoo.com.tw (Y.-H.H.); mindymhguo@yahoo.com.tw (M.M.-H.G.); trentlo@adm.cgmh.org.tw (M.-H.L.); 2 Department of Pediatrics, Chiayi Chang Gung Memorial Hospital, Chiayi 613016, Taiwan; atlas8@cgmh.org.tw; 3 Beijing Institute of Technology, School of Life Science, Beijing 100081, China; jinyuli99@126.com; 4 KU School of Nursing, Nursing Associate Tech Adult Inpatient Psych KU Strawberry Hill Campus, Kansas City, KS 66101, USA; deniz.yeter@gmx.de; 5 Graduate Institute of Clinical Medical Sciences, College of Medicine, Chang Gung University, Kaohsiung 83301, Taiwan; 6 Department of Respiratory Therapy, Kaohsiung Chang Gung Memorial Hospital, Kaohsiung City 83301, Taiwan

**Keywords:** Kawasaki disease, mercury, sodium

## Abstract

The risk of ethnic Kawasaki disease (KD) has been proposed to be associated with blood mercury levels in American children. We investigated the blood levels of mercury in children with KD and their association with disease outcome. The mercury levels demonstrated a significantly negative correlation with sodium levels (*p* = 0.007). However, data failed to reach a significant difference after excluding the child with blood mercury exceeding the toxic value. The findings indicate that KD patients with lower sodium concentrations had a remarkably higher proportion of intravenous immunoglobulin (IVIG) resistance (*p* = 0.022). Our patients who had lower mercury levels (<0.5 μg/L) had more changes in bacille Calmette-Guerin. Mercury levels in 14/14 patients with coronary artery lesions and 4/4 patients with IVIG resistance were all measured to have values greater than 1 μg/L (while average values showed 0.92 μg/L in Asian American children). Mercury levels had no correlations with IVIG resistance or coronary artery lesion (CAL) formation (*p* > 0.05). CAL development was more common in the incomplete group than in the complete KD group (*p* = 0.019). In this first report about mercury levels in KD patients, we observed that the juvenile Taiwanese had higher mercury concentration in blood compared to other populations.

## 1. Introduction

Kawasaki disease (KD) has become more prevalent in recent years and is the most common cause of pediatric acquired heart diseases [1]. A global disorder, KD has varying incidence rates that primarily reflect the different ethnicity of the populations [2]. In addition to the well-known highest incidence in Japan, the incidence of KD is high in Korea and Taiwan, while it is low in North America and European countries [3]. The pathogenesis of KD is multi-factorial. Being an Asian is a renowned risk factor for KD in the United States [4,5]. Our previous study suggested that mercury levels were also associated with admission for KD in different ethnic American populations [5]. Furthermore, abnormally elevated mercury levels have been associated with infantile acrodynia, a juvenile mercurial illness that may mimic KD [6]. In fact, infantile acrodynia and KD have some similarities in clinical, historical, and epidemiological features. Thimerosal is an organic form of mercury used in several pediatric vaccines, including bacille Calmette-Guerin (BCG) [7]. The American Academy of Pediatrics suggested removing thimerosal in vaccines in 1999 [8]. Carriers of the single nucleotide polymorphism inositol 1,4,5-triphosphate kinase C (ITPKC) are more susceptible to KD, as well as thimerosal-induced sensitization to IP3 receptors through the production of oxidative stress, thus leading to the raised release of intracellular calcium [6,9,10,11,12]. The genetic variation of ITPKC may be associated with the BCG vaccine scar of Taiwanese patients with KD [13]. The highest immunization rates are in Taiwan and Japan due to endemic tuberculosis, where reactions at the BCG site have been recognized as a significant and common finding in KD patients [14,15]. BCG, hepatitis B virus, Japanese encephalitis or diphtheria and tetanus toxoid with acellular pertussis, Inactivated polio and Haemophilus influenzae type b vaccines do not contain thimerosal currently in Taiwan. BCG preparations differ among countries. In Japan, the BCG manufacturing process does not require the use of thimerosal.

Oxidative stress can be stimulated via contacting mercury in the form of methylmercury crossing the blood–brain and placental barriers, which may occur through fish consumption, vaccination, or environmental contamination by disposed personal computers or local industrial emissions in Taiwan [16]. Methylmercury, an organic form of mercury, is a neurotoxicant associated with impaired neurologic development. Another inorganic form released into the air, which develops into atmospheric contamination and is accumulated in the environment, can be converted to methylmercury [17]. Children are an extremely vulnerable group, and 70–95% of total mercury levels in whole blood is in the form of methylmercury binding to hemoglobin, which can be used to reflect methylmercury exposure [17,18].

In 2015, Sharma et al. summarized that mercury concentration in the peripheral blood was highest in Africa, followed by South America or Asia and lowest in Europe and North America [19]. In our previous study, we found that mercury concentration in the peripheral blood was highest in KD-vulnerable Asian American children and lowest in Caucasian Americans [5]. However, no study has yet measured blood mercury levels in KD groups. The aim of this study was to investigate peripheral blood mercury levels in patients with KD and their association with disease outcome.

## 2. Materials and Methods

### 2.1. Collection of Blood Samples for Blood Mercury Estimations

This study was developed in the Kaohsiung Chang Gung Memorial Hospital and carried out between 2016 and 2020. We enrolled a total of 85 KD patients. Subjects consisted of 48 boys (56.47%) and 37 girls, aged 0 months to 6 years. Ethical approval for the study was obtained from the Chang Gung Medical Foundation Institutional Review Board (IRB 201601736A3). KD was diagnosed in line with the American Heart Association diagnostic guidelines for KD [20].

Total blood routine lab data were determined prior to intravenous immunoglobulin (IVIG) infusion. Echocardiography was performed in the acute stage of KD as previously described in our report [21]. Patients experiencing persistent or recurrent fever (≥38 °C) 48 h after the initiation of the first IVIG treatment were recognized as the IVIG resistance group. Patients with complete KD defined when a child had four or more of the diagnosis criteria [22]. In this study, coronary artery lesions (CAL) were verified as an internal coronary artery diameter ≥3 mm in KD patient younger than five years old and ≥4 mm in those more than five years old [23]. Two g/kg of IVIG were standardly prescribed over 12 h.

We collected 3 mL whole blood samples in K2EDTA tubes (Becton, Dickinson and Company, New Jersey USA). The tubes were placed at 4 °C for the evaluation of total Hg later. Clotted whole blood was disallowed and rejected. The detection limit of the assay was 0.2 μg/L. To analyze mercury levels in the whole blood, the Inductively Coupled Plasma-Mass Spectrometry (ICP-MS) technique on PerkinElmer NexIon 300 (Waltham, MA, USA) equipment was used and reported as micrograms per liter (μg/L). The laboratory conducted blanks for quality control daily.

### 2.2. Statistical Analyses

Statistical tests using the Statistical Package for Social Sciences (SPSS) (IBM SPSS Inc., Chicago, IL, USA) were analyzed. We carried out non-parametric tests for the statistical analyses according to variable distributions as follows: the Mann–Whitney *U* test for continuous values to calculate the numeric values, the chi-square test was for comparing categorical values, and Pearson’s correlation to estimate the relationships between blood biochemistry and mercury levels. All *p*-values were 2-sided with alpha values less than 0.05 considered statistically significant.

We identified a comparable concentration of total mercury in the whole blood of populations susceptible to KD in the literature published in English. An extensive search of children and adolescents under the age of 18 years old as target groups was carried out on PubMed and included hand-searched references and screened citation lists. The keywords were “mercury AND blood AND child”. We identified relevant articles using the mercury measurement unit, μg/L. We excluded those reports without juvenile blood mercury data. This search turned out 840 peer-reviewed articles reported before 20 February 2020. We ultimately chose a total of six peer-reviewed studies including relevant age and our current reports for data extraction [5,17,24,25,26,27].

From each eligible study, we found the central value (arithmetic mean, geometric mean, or median) of mercury data. In surveys that only recorded the arithmetic means of blood mercury, we assumed that the data were normally distributed and took the arithmetic mean to be as a representative of central value as median. In the further steps of data treatment and analysis, the (Maximum—Minimum)/4 formula was used to estimate the standard deviation [28]. We subsequently tested the difference between the juvenile sub-groups (e.g., school-aged and preschool-aged children), using t-test and Bonferroni’s correction analyzed by MedCalc Statistical Software (MedCalc Software bvba, Ostend, Belgium).

## 3. Results

### 3.1. Clinical Characteristics of Total KD Patients

The mean age of KD patients (*n* = 85) was 1.90 ± 0.14 years, and the male-to-female ratio was 1.30 (48:37). Among the total patients, 68 presented with complete KD and 17 with incomplete KD. The prevalence rates of the clinical symptoms of the eyes, oral changes, polymorphous rashes, extremity edema, palpable cervical lymphadenitis, and BCG scar reactivation were 94.1%, 90.6%, 87.1%, 87.1%, 38.8%, and 63.6%, respectively. Fourteen children (16.5%) demonstrated echocardiographic abnormalities in the acute stage. CAL development was more common in the incomplete group than in the complete KD group (35.29% and 11.76%, respectively, *p* = 0.019). Four children did not respond to initial IVIG treatment. All four IVIG-resistant patients had BCG scar activation, polymorphous rash, swelling of extremities, conjunctival congestion, and changes in oral mucosa and belonged to the complete KD group, but this finding did not reach statistical significance.

### 3.2. Association of Mercury Levels with the Clinical Features and Outcomes of Kawasaki Disease

The mean mercury was 4.15 ± 0.37 (standard error) μg/L. Of the 79 eligible KD patients, mercury levels in the acute phase prior to administering initial IVIG ranged from 0.4 to 19.4 μg/L, with a median of 3 μg/L. The mercury levels of six KD patients were undetectable. We excluded undetected data in our analysis for the continuous mercury variable (*n* = 79) but included undetected data in the analysis for categorical mercury variable (*n* = 85). No statistically significant difference was found in mercury levels (*p* = 0.448) between male and female KD patients prior to IVIG treatment. Furthermore, no correlation was observed in age or pre-IVIG mercury levels (*r* = 0.096, *p* = 0.402). Pre-treatment mercury levels > 1 μg/L were detected in all of the KD patients with CAL or IVIG resistance without a significant difference. For patients with mercury > 1 μg/L, we observed a borderline significant difference between CAL and non-CAL groups (*p* = 0.058). No significant difference was found in mercury levels between IVIG-responsive and IVIG-resistant groups (median: 3 (interquartile range 1.80–5.70) μg/L vs. 5.4 (1.65–8.93) μg/L, *p* = 0.553), nor in patients with complete and incomplete KD (2.9 (1.80–5.68) μg/L vs. 3.5 (1.5–6.40) μg/L, *p* = 0.900). No significant association was observed between mercury levels and BCG site reaction or clinical features of KD presented (Table 1). Unexpectedly, induration and erythema on the BCG site were significantly less frequent in KD patients with mercury > 0.5 μg/L (*p* = 0.045). Age at onset was significantly lower in the BCG change group (1.33 ± 0.08, and 2.78 ± 0.32 years-old, respectively; *p* < 0.001). The blood mercury concentration in the five age and gender matched non-KD control group (4.42 ± 1.17 μg/L) and in children with KD were similar (*p* = 0.569). Patients in the control group suffered from acute infections, including four bacterial infections and one child hospitalized for herpetic gingivostomatitis.

### 3.3. Association of Mercury Levels with the Laboratory Characteristics of Kawasaki Disease

**Figure 1:**
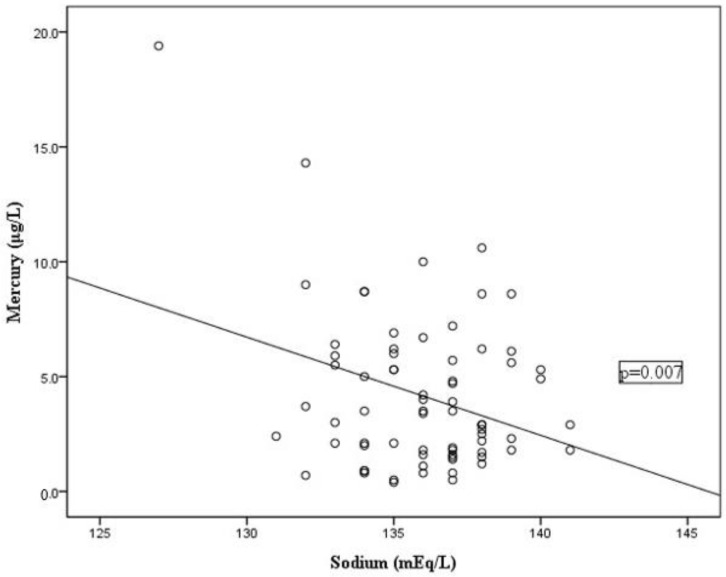
Scatter plot and correlation analysis between blood mercury and sodium. Blood mercury showed a strong negative correlation with sodium (*r* = −0.318, *p* = 0.007).

With Pearson’s correlation coefficient, mercury levels showed a strong association with sodium (*r* = −0.318, *p* = 0.007) but no statistically significant correlation with leukocyte count, percentage of neutrophil, lymphocyte, monocyte, eosinophil, basophil, platelet count, aspartate transaminase, alanine transaminase, or C reactive protein. However, data failed to reach a significant difference after excluding the child with blood mercury exceeding the toxic value above 15 μg/L. A total of seven children with mercury data were missing blood sodium concentrations. Figure 1 shows the associations between mercury and sodium in patients with new-onset KD.

After obtaining a positive finding, we further investigated the association between the ratio of blood mercury to sodium and disease outcome. However, the ratio of blood mercury to sodium between CAL and non-CAL groups failed to reach a significant difference (*p* > 0.05). No significant difference in the ratio of blood mercury to sodium was observed between the IVIG response and resistant groups.

The relationship between mercury levels and laboratory data values is presented in Table 2. Lower sodium levels (<135 mEq/L) were observed in the IVIG-resistant group compared to the responsive group (*p* = 0.022).

Paired blood samples (*n* = 14) were obtained from patients with KD before and after treatment with IVIG. The tests revealed no difference in mercury levels between patients with KD before and after IVIG (2.25 (0.775–5.5) μg/L vs. 2.5 (1.20–5.75) μg/L, *p* = 0.177).

### 3.4. Total Mercury Levels in Whole Blood for Different Populations

Elevated ethnic KD risk was especially associated with higher mercury in blood among ethnic African, Asian, Caucasian, and Hispanic children in the United States. Our study was further replicated in the other ethnic populations most susceptible to KD. The highest blood mercury levels were in Taiwanese children (7–12 years, mean ± standard deviation: 5.33 ± 3.29 μg/L in 2005–2008) from one of the National human biomonitoring, followed by the Japanese population in Asahikawa (9 to 10 years, 4.55 ± 2.49 μg/L in 2008–2009), Korean population (8 to 18 years, 2.12 ± 0.99 μg/L in 2008–2011), and American population (6 to 11 years, 0.41 ± 1.067 μg/L in 2007–2008) [29]. Similar to mercury levels in school children, blood mercury levels in preschool children displayed the highest blood mercury levels in Taiwanese KD children, followed by Korean (0 to 7 years, 2.05 ± 1.06 μg/L in 2008–2011), and were lowest in Asian American and American children (1 to 5 years, 0.92 μg/L; 0.45 μg/L in 2011–2012). The mercury levels in Chinese blood were lower than those in in Korean blood (0 to 6 years, 1.10 μg/L in 2013–2015) but higher than those found in America. All *p*-values were calculated using the t-test with Bonferroni’s correction and had values less than 0.05 (Table 3).

## 4. Discussion

To the best of our knowledge, we are the first to determine blood mercury levels in KD patients and all the patients in this study are from Taiwan. After actually measuring the mercury concentration in KD patients’ blood, we found that mercury concentration was not related to either clinical manifestations or disease prognosis. Presumably, the cause may be that KD patients primarily presented with blood mercury levels < 15 μg/L (98.82%), which are considered normal and subtoxic [30,31]. However, we still need to watch out for KD patients with blood mercury levels greater than 1 μg/L. A BCG site change in patients with KD is considered to be due largely to the younger onset age and shorter interval from the BCG vaccination [32]. Furthermore, we demonstrated that KD patients who had lower mercury levels (<0.5 μg/L) were more likely to have changes in BCG.

These results suggest that blood mercury levels were negatively correlated with sodium levels. Carter et al. reported a boy with acute mercury toxicity was complicated by hyponatremia [33]. Similar to this report, our research included one pediatric patient with mercury poisoning and hyponatremia. According to the results of mercury concentration compared between KD patients and the control group and before and after IVIG administration, mercury concentration may be more relevant to mercury exposure than KD. However, we did not clarify his contacting environment of mercury condition. Observations of rats have suggested that chronic oral exposure to mercury results in decreased sodium levels [34]. However, plasma sodium did not decrease in juvenile male chickens exposed to mercury through drinking water [35]. Chronic methylmercury poisoning usually causes neurological complications not hyponatremia. Since humans used to apply mercury as a diuretic before the proper medicine was developed, understanding the relationship between mercury and sodium in human blood is not hard. Manifestation of renal toxicity with proteinuria resulted from glomerular and renal tubular damage by mercury [36].

Exposure to environmental toxins like mercury is known to have lasting adverse effects on the cardiovascular system, such as coronary heart disease in adults [37]. However, we found no relationship between blood mercury levels and CAL. The possible reason for this result is too few case numbers in our study or different reaction by adults and children to mercury.

A national survey for mercury concentration in blood suggests the higher levels in Asia, while the population groups from the United States have the lower levels of total mercury, consistent with the high incidence of KD in Japan, Korea, China and Taiwan and its low incidence in North America. Although the instruments used to measure mercury concentration in studies from the United States (ICP-MS), Taiwan (ICP-MS), South Korea (Direct Mercury Analyzer), China (Direct Mercury Analyzer), and Japan (cold vapor atomic absorption spectrometry) were different, the determination of mercury concentration in fish found by ICP-MS was not significantly different than the one obtained by Direct Mercury Analyzer [38]. The mercury concentrations measured using ICP-MS and cold vapor atomic absorption spectrometry were in good agreement for environmental samples [39]. However, the epidemiological data of large-scale mercury concentrations in preschool children as a control group is lacking in both Taiwan and Japan, and mercury concentrations in KD patients of other races are also lacking. Another limitation was no ability to assess or restrict patients’ diets. The juvenile Taiwanese population presented the highest levels of mercury levels in blood compared to the Japanese, Korean, and American populations. Under the same race, genetic background and contact environment, not only in juvenile Taiwanese individuals, both Lee and Liu et al. demonstrated the remarkably high blood total mercury in adult Taiwanese individuals (average 13.8 and 9.64 μg/L) [16,40]. Small-scale sampling shows that the mercury concentration in the urine of elderly Taiwanese people was 1.2 μg/L [41]. This value reflected the long-term exposure of mercury, which was relevant to dietary exposure to methylmercury [42]. The mercury concentration in children’s urine was 1 μg/L, which was much lower than the health-based guidance values in urine (7 μg/L) [43,44].

The consumption of seafood is important in general Japanese and Taiwanese food, regardless of age. In addition to the fact that fish intake may be a main source of mercury, industrial emissions to the soil’s surface, atmospheric pollution from overseas, distinct living region, age, and races were also considered as factors affecting blood mercury levels [16]. Mercury cannot be removed from the contaminated soil [45]. Twenty years after being contaminated with mercury, the mercury concentration in the blood of residents living near an abandoned chloralkali factory still remains high [46]. Consumption with large oily marine fish is very important for the mercury accumulation in Taiwanese individuals [16]. High air mercury concentrations since 2000–2008 in Asia and Taiwan also contributes to the lasting high concentration of Taiwanese blood mercury [47]. To protect vulnerable populations, especially KD-susceptible children in Taiwan, it is important to be aware of any pollution in locally caught or raised and consumed fish contaminated with mercury or methylmercury.

## 5. Conclusions

In terms of national comparisons, we reported that children in Taiwan displayed the higher concentrations of mercury. This finding highlights the importance of studying blood mercury concentrations in Taiwanese children and also mercury levels in KD patients. Large-scale comprehensive studies are needed to protect children’s health, reduce and avoid mercury exposure.

## Figures and Tables

**Table 1:** Comparison of mercury levels with different clinical symptoms in Kawasaki disease patients.

	^1^ Mercury Levels in Negative Presentation μg/L	^1^ Mercury Levels in Positive Presentation μg/L	*p*-Value
Changes in oral mucosa	3.25 (0.95–8.33)	3.00 (1.80–5.70)	0.922
Conjunctivitis	3.00 (1.55–6.70)	3.15 (1.78–5.75)	0.884
Palpable cervical lymphadenopathy	3.60 (1.75–6.08)	2.90 (1.80–5.30)	0.448
Edema of extremities	3.20 (1.95–6.10)	3.00 (1.70–5.80)	0.947
Skin rash	3.40 (1.80–5.70)	3.00 (1.75–5.95)	0.825
BCG ^2^ scar reactivation	2.90 (1.60–5.30)	3.40 (1.70–6.15)	0.696
Incomplete KD ^3^	2.90 (1.80–5.68)	3.50 (1.50–6.40)	0.900

^1^ Data presented by median (interquartile range). ^2^ BCG, bacille Calmette-Guerin. ^3^ KD, Kawasaki disease.

**Table 2:** Correlations between blood mercury and sodium levels in patients with acute Kawasaki disease.

Variable	Blood Mercury	
	Pearson *r*	*p*-Value
Sodium	−0.318	0.007 *
Leukocytes	0.016	0.892
Percentage of neutrophil	0.076	0.506
Percentage of lymphocyte	−0.109	0.340
Percentage of monocyte	−0.053	0.642
Percentage of eosinophil	−0.024	0.836
Percentage of basophil	−0.011	0.927
Platelet count	−0.041	0.721
Aspartate transaminase	0.095	0.406
Alanine transaminase	0.057	0.618
C reactive protein	0.061	0.596

* Correlation is significant at the 0.05 level (2-tailed).

**Table 3:** Average values for blood mercury in children.

Values for Blood Mercury in School-Aged Children				
	μg/L		μg/L	*p*-Value
Taiwan	5.33	Japan	4.55	0.0025
Japan	4.55	Korea	2.12	<0.01
Korea	2.12	America	0.41	<0.01
**Values for Blood Mercury in Preschool-Aged Children**				
Taiwanese with Kawasaki disease	4.15	Korea	2.05	<0.01
Korea	2.05	China	1.10	<0.01
China	1.10	America	0.45	<0.01
